# Magnitude and predictors of antiretroviral treatment failure among HIV‐infected children in Fiche and Kuyu hospitals, Oromia region, Ethiopia: a retrospective cohort study

**DOI:** 10.1002/prp2.296

**Published:** 2017-01-25

**Authors:** Seid Yassin, Gebremedhin Beedemariam Gebretekle

**Affiliations:** ^1^ International Center for AIDS Care and Treatment ProgramAddis AbabaEthiopia; ^2^ Department of Pharmaceutics and Social PharmacySchool of PharmacyCollege of Health SciencesAddis Ababa UniversityAddis AbabaEthiopia

**Keywords:** ART, clinical, Ethiopia, HIV/AIDS, immunologic, pediatrics, treatment failure

## Abstract

The objective of the study was to assess the magnitude and predictors of first‐line antiretroviral treatment failure among HIV‐infected children. A retrospective cohort study was conducted between January 2006 and December 2015. All pediatric patients of <15 years old and who took at least 6 months of ART in Fiche and Kuyu hospitals of Oromia region, Ethiopia were included in the study. Collected data were entered and analyzed using SPSS version 20. Multivariable logistic regression was employed to identify predictors of treatment failure. Data of 269 children were analyzed and majority (53.9%) of the children were males with a mean age of 9.8 ± 3.64 years. Based on the two WHO criteria, overall ART failure was found to be 51 (18.8%), of which 33 (12.26%) had clinical and 18 (6.69%) had immunologic failures. The mean time to the detection of treatment failure was 41 ± 24.96 months. Children's age between 6 and 9 years (AOR = 0.26, 95% CI: 0.09, 0.72) was protective toward treatment failure, while lost to follow‐up (AOR = 7.54, 95% CI: 2.35, 24.16), died (AOR = 22.22, 95% CI: 3.75, 131.54), transferred out (AOR = 3.34, 95% CI: 1.41, 7.87), suboptimal adherence to ART (AOR = 4.85, 95% CI: 1.82, 12.93), baseline CD4 count of <50 cells/mm^3^ (AOR = 4.28, 95% CI: 3.49, 5.9), and WHO advanced clinical stage (AOR = 2.46, 95% CI: 1.14, 5.31) were found to be predictors of treatment failure. The study revealed that the treatment failure is high and the mean time to develop treatment failure is short. The predictors for treatment failure were suboptimal adherence, lost to follow‐up, transferred out, initial CD4 count <50 cells/mm^3^, initial WHO stages 3 and 4. On the other hand, being in the age of 6–9 years is protective from developing treatment failure as compared to the other age category.

**Abbreviations**
AIDS: acquired immune deficiency syndrome
ART: antiretroviral treatment
ARV: antiretroviral
CD4: cluster of differentiation 4
CDC: Center for Disease Control and Prevention
DBS: dried blood spot
HIV: human immunodeficiency virus
IPT: isoniazid prophylaxis
OI: opportunistic infection
PMTCT: prevention of mother to child transmission
WHO: World Health Organization

## Introduction

Although initially acquired immune deficiency syndrome (AIDS) was discovered among a certain vulnerable populations, it later became a worldwide pandemic. In 2015, there were 2.1 million new human immunodeficiency virus (HIV) infections worldwide, adding up to a total of 36.7 million people living with HIV (WHO, 2016). In the early decades of the HIV epidemic, children infected with HIV had relatively little hope of survival into adolescence as there was no efficient therapy to stop the virus. With the development of safe and effective medicines, however, HIV‐positive children and adolescents now survived much longer and have healthier lives (Osterholzer 2013).

Even with a lot of efforts in place to control the disease, it remains a major global public health challenge. There are 2.1 million new HIV infections that occurred globally and majority of cases are in sub‐Saharan African countries. HIV infection among children constitutes 7.5% of people living with HIV worldwide and 14.4% of total HIV/AIDS deaths. About 50% of children living with HIV/AIDS die before the age of 2 years as a result of recurrent opportunistic infections such as pneumonia, diarrhea, malnutrition, and malaria (WHO, 2010).

At the end of 2015, there were 17 million people on ART and the global coverage reached to 46% (WHO, 2016). On the other hand, WHO reported that there are 500,000–800,000 patients on ART follow‐up worldwide who are in need to be switched to second‐line ARV treatment (Boyd 2010). A study done in India showed that 13.95% of patients had immunological failure who were in need of switching to second‐line regimen (Prabhakar et al. 2011). Another study in South Africa found that only 2% of the patients taking first‐line ART were switched to second‐line ARV, but authors revealed that results could increase if appropriate clinical, immunologic, and viral load testing were done. Besides, virology treatment failure ranges from 8% to 17% for patients on ART care, but they found that there was delay in assessing, managing, and shifting first‐line ARV failures (Fox et al. 2012).

Ethiopia is one of the top five African countries having total of 765,500 people living with HIV/AIDS (Prabhakar et al. 2011). First evidence of HIV/AIDS in Ethiopia was found in 1984; it claims millions of human life and resulted in millions of children without family. The 2014 EDHS report showed that prevalence of HIV/AIDS dropped to 1.14%, being high in urban (3.81%) and low in rural (0.57%). The country had 367,000 HIV patients on ART, of which 23,400 were <15 years old (Federal Ministry of Health [FMOH], 2014).

In Oromia region (one of the 11 regions in Ethiopia where our study hospitals are located), free ART service provision was started in three hospitals since 2005 and later decentralized to other hospitals and health centers. During the program initiation, general medical practitioners were the sole service providers, but later the government started a task shifting to other health professionals (health officers and clinical nurses) so as to increase access. In June 2014, the region had a total of 194,370 HIV‐positive patients and 115,334 of them were on ART follow‐up, of these 15,586 were <15 years old (Bacha et al. 2012).

Even if many HIV‐positive clients accessed ART, there is a growing problem of treatment failure. In resource‐limited settings, treatment failure is a growing problem that threatens to undermine much of the work that has been done. Pediatric cases of ART failure rates ranged from 19.3% to over 32% (Workneh et al. 2009). Retrospective cohort study done in Uganda and Mozambique also found that ART failure rate was 29% (Teshome and Bacha 2015) and other retrospective cohort conducted in Jimma University hospital reported 11.5% immunologic treatment failure (Workneh et al. 2009).

Similar retrospective cohort study done in four public hospitals in Addis Ababa showed that of the 1186 pediatric patients on ART, 14.1% of the children had evidence of first‐line ART failure, of which 70 (5.9%) had clinical treatment failure, 79 (6.7%) immunologic failure, and 18 (1.5%) developed both immunologic and clinical failure (FMOH, 2014). A study conducted by Yimer and Yalew (2015) also showed that the extent of ART failure in the private health facilities in Ethiopia is high (20.4%).

There are various risk factors described in different studies; the sociodemographic factors (e.g., age), baseline clinical factors (e.g., baseline CD4 count and WHO clinical stage), drug–drug interactions, drug side effects, drug toxicity, or inadequate adherence to treatment are some of the factors associated with treatment failure (Workneh et al. 2009; Prabhakar et al. 2011; Bacha et al. 2012; Fox et al. 2012; Teshome and Bacha 2015; Yimer and Yalew 2015). Nevertheless, there is no study for pediatric patients in North Shewa of the Oromia region indicating the magnitude and predictors of treatment failure. So the aim of this study was to assess the magnitude and predictors of treatment failure among HIV‐infected pediatric patients (with age ≤15 years) on ART and to provide information to optimize treatment outcomes.

## Materials and Methods

### Study area

This study was conducted in North Shewa Zone of Oromia region which is located 112 km from the capital city of Ethiopia, Addis Ababa. The zone has a total population of 1,508,741, of which 755,218 (51%) were male. It has 14 woreda health offices, and ART service is being provided by 15 ART sites (13 ART health centers, 2 hospitals). Besides, there are 15 prevention of mother to child transmission (PMTCT) only sites providing option B+ service in the zone. Since the initiation of the program, a total of 7075 patients started ART, and of them 5174 were on ART, as of December 2015. Fiche and Kuyu hospitals are among high client load ART facilities in Oromia region, and they started provision of free ART services in 2005 (FMOH, 2015; North Shewa Zonal Health Department, 2015).

### Study design

Institution based retrospective cohort study design had been used to retrieve relevant information from HIV‐infected pediatric patients' medical chart. Children seen at these centers were followed based on the national guideline which recommends that children are evaluated 2 weeks after initiation of ART, every month for the next 2 months, and every 3 months afterward. Pediatric patients <15 years old, who were on ART regimen between January 2005 and December 2015, and who had a minimum of two follow‐up visits with at least one visit 6 months postinitiation of ART were included in the study. Children who took ART only for PMTCT were excluded.

Data were collected using a pretested data abstraction format prepared based on the national guidelines, ART follow‐up forms, and by reviewing similar literatures, from intake and follow‐up forms designed for pediatric ART patients in Ethiopia. The data collection was designed to capture information on age, gender, school enrollment status, weight for age, duration of follow‐up, clinical and laboratory data (WHO stage, opportunistic infections, and CD4 count), ART regimen, prophylaxis, and adherence level in pediatric patients. Caregivers‐related data were also retrieved from the record.

Two diploma level holders of ART clinic data entry clerks were recruited as data collectors. The data collectors attended a 1 day training focused on the aim of the study and detailed review of the tool. In addition to the practical training, adequate supervision and follow‐up were done by the supervisor to maximize quality of the data collected.

### Data analysis

The collected data were then coded, entered, cleaned, and analyzed using SPSS version 20. Simple descriptive statistics including mean, percentage, and standard deviations were computed to summarize categorical variables. Moreover, multivariable logistic regression was performed to explore possible associations between the dependent variable (first‐line ART failure) and the independent variables. Association between the outcome and the independent variables was taken as significant at *P *< 0.05.

The study used the standard definition of immunologic failure which is defined as developing or returning to the following age‐related immunological thresholds after at least 24 weeks on ART, CD4 count of <200 or <10 for child 2–5 years, and CD4 count <100 for a child 5 years or older. Clinical failure is also considered for a child with appearance or reappearance of WHO clinical stage 3 or 4 disease at least 24 weeks after treatment initiation (FMOH, 2014).

In Ethiopia viral load testing is not available in routine practice settings; hence, treatment failure in this study is determined based on the two WHO criteria (immunological and clinical failures). In this cohort, the mean time to detection of treatment failure indicates the time between ART initiation and detection of failure of first‐line ART. Time to switch to second‐line ART regimens represents the time between detection of first‐line treatment failure and the time of initiation of second‐line drugs. Suboptimal adherence is defined in terms of total missed appointments described by more than 3 days per month (FMOH, 2014; Bacha et al. 2012).

### Ethical considerations

Ethical approval was sought from the Ethics Review Committee of Africa Medical College and Oroima Regional Health Bureau. Permission letter was also granted from North Shewa zonal health department and both hospitals. Patient cards were coded and collected data were locked in a lockable cabinet in order to maintain confidentiality of the information obtained.

## Results

### Sociodemographic characteristics of respondents

A total of 269 study participants medical charts were reviewed, of them 118 and 151 pediatric patients had ART follow‐up in Kuyu and Fiche hospitals, respectively. More than half (53.9%) of patients were males and majority (56.8%) of the pediatrics were within the age of 10–15 years (Table 1). The mean age of the children was 9.8 ± 3.64 years, ranged from 1 to 14 years. The mean ART clinic follow‐up period of the current study was 43.17 ± 9.60 months with minimum 6 months and maximum 124 months of follow‐up.

**Table 1:** Sociodemographic characteristics of pediatric patients on ART in Fiche and Kuyu hospitals followed from 2006 to 2015, North Shewa zone, Oromia region, Ethiopia (*N* = 269)

Patient characteristics	*N* (%)
Gender	
Male	145 (53.9)
Female	124 (46.1)
Age (years)	
<5	34 (12.6)
5–9	82 (30.4)
10–15	153 (56.8)
School enrollment	
Enrolled	205 (76.2)
Not enrolled	64 (23.8)
Parent status	
Both alive	148 (55.0)
One parent alive	71 (26.4)
Both dead	50 (18.6)
Employment status of primary caregiver	
Employed	107 (39.8)
Not employed	162 (60.2)
Primary caregiver of the child	
Mother	185 (68.8)
Father	34 (12.6)
Guardian/relative	50 (18.5)

Record of school enrollment status of study participants indicated that majority (76.2%) of them had history of school enrollment records. Regarding their family status, 148 (55%) of them reported that both of their parents were alive, 71 (26.4%) had one parent alive, and 50 (18.6%) of the pediatrics claim that both parents died. The study also showed that most (68.8%) of the study participants were accompanied by their mother as their primary caregiver, followed by fathers, 33 (12.3%). Concerning the employment status of their primary caregivers, more than two third (60.2) of the caregivers were not employed (Table 1).

### Medical and clinical follow‐up history of participants

From the total cohort of 269 study participants, the majority (73.6%) of them were on ART follow‐up at the time of study, 19 (7.1%) were reported as lost, 9 (3.3%) dead, and 43 (16.0%) transferred out. The study also revealed that PMTCT coverage was very low, where only 16 (5.9%) of them had documented provision of NVP prophylaxis during delivery and postnatal period. Isoniazid prophylaxis (IPT) was provided to 158 (58.7%) of the pediatrics, but the rest (41.3%) of the participants did not received IPT at least once (Table 2).

**Table 2:** Clinical and treatment data history of pediatric patients on ART in Fiche and Kuyu hospitals followed from 2006 to 2015, North Shewa zone, Oromia region, Ethiopia (*N* = 269)

Patient characteristics	*N* (%)
Status at last visit	
Lost	19 (7.1)
Died	9 (3.3)
Transferred out	43 (16.0)
On ART	198 (73.6)
PMTCT intervention	
Yes	16 (5.9)
No	253 (94.1)
Eligibility criteria for ART initiation	
Clinical	100 (37.2)
Immunologic	48 (17.8)
Both (clinical and immunological)	117 (43.5)
DBS result	4 (1.5)
Initial ARV regimen	
4a (d4t + 3TC + NVP)	124 (46.1)
4b (d4t + 3TC + EFV)	8 (2.9)
4c (AZT + 3TC + NVP)	98 (36.4)
4d (AZT + 3TC + NVP)	19 (7.2)
Others^a^	20 (7.4)
Last 6 months adherence	
Optimal	227 (84.4)
Suboptimal	42 (15.6)
Initial WHO stage	
Stage 1	59 (21.9)
Stage 2	53 (19.7)
Stage 3	145 (53.9)
Stage 4	12 (4.5)
Duration of follow‐up (months on ART)	
<11.9	40 (14.9)
12–23.9	49 (18.2)
24–35.9	47 (17.5)
36–47.9	27 (10.0)
>48	106 (39.4)
Baseline CD4 count (*n* = 246)	
<50 cells/mm^3^	8 (3.3)
50–200 cells/mm^3^	51 (20.7)
201–500 cells/mm^3^	92 (37.4)
>500 cells/mm^3^	95 (38.6)
Disclosure of HIV positive status	
Yes	16 (5.9)
No	253 (94.1)
Initial weight for age (*n* = 208)	
<3rd centile	93 (44.71)
>3rd centile <97 centile	113 (54.33)
>97 centile	2 (0.96)
Missed appointment days (*n* = 47)	
<8 days	3 (6.4)
8–30 days	33 (70.2)
>30 days	11 (23.4)
Was treatment failure developed	
Yes	51 (18.96)
No	218 (81.04)
Time to development of treatment failure (*n* = 51)	
<24 months	7 (13.7)
24–36 months	11 (21.6)
>36 months	33 (64.7)
Time taken to initiate second‐line ARVs regimen after treatment failure (*n* = 12)	
<30 days	9 (75.0)
≥30 days	3 (25.0)
Opportunistic infection after 6 months	
Yes	34 (12.64)
No	235 (87.36)
Type of opportunistic infection after 6 months (*n* = 34)	
Extrapulmonary tuberculosis	10 (29.4)
Diarrhea	5 (14.7)
Oral thrush	3 (8.8)
Others^b^	16 (47.1)

a Others include 5e‐TDF/3TC/EFV, 5g‐ABC/3TC/EFV, 5h‐ABC/3TC/NVP.

b Others include *Pneumocystis carinii* pneumonia, toxoplasmosis of the brain, cryptococcosis, oral candidiasis, HIV wasting syndrome.

From the 269 pediatrics who were eligible for ART initiation, 100 (37.2%) of the ART were initiated by advanced WHO clinical staging criteria, 48 (17.8%) due to low CD4 count, 117 (43.5%) by both immunologic and clinical criteria. During the initiation of ART, 59 (24.0%) of study participant were found to be in advanced immunodeficiency state with CD4 <200 cells/mm^3^ and of these, 8 (3.3%) were at severe immunological stage with CD4 count <50 cells/mm^3^. Record of their initial WHO stage also revealed that more than half (53.9%) of the pediatrics were at WHO clinical stage 3. The choice of ART regimen for pediatric patients was compared and accordingly, higher proportion (89.7%) of initial regimen prescribed at baseline included nevirapine as a combination. A d4t‐3TC‐NVP regimen covered 124 (46.1%) and d4t‐3TC‐EFV regimen covered 8 (2.9%) of the total prescribed ARV regimen. Zidovudine (AZT) with NVP was prescribed as initial regimen to 98 (36.4%) of the pediatric patients. However, first‐line ARV regimen substitution was done for 131 (48.7%) to other alternative group first‐line ARV drugs due to side effect, severe toxicity, and opportunistic infection, and most of them taking 4a (d4t+3TC+NVP).

Adherence to ART for the last 6 months of follow‐up was assessed and the study showed that majority (84.4%) of the study participants had optimal adherence and 48 (15.6%) of the pediatrics had suboptimal adherence. The number of missed appointment days was also found to be high where more than 9 (93.6%) of the 10 study participants had missed appointments for 8 days or more. The HIV‐positive status of the study participants was not disclosed to majority, 253 (94.1%), of the pediatric patients (Table 2).

Baseline nutritional assessment using weight for age was done employing Center for Disease Control and Prevention (CDC) standard growth curve. The study revealed that most of them were malnourished, where 93 (44.71%) of them were severely malnourished and 113 (54.33%) of the children were moderately malnourished. Similar anthropometric measurement was sought for the last visits and 57 (21.2%) and 128 (47.6%) of the pediatrics were found to be severely and moderately malnourished, respectively. Of the children, 34 (12.64%) developed opportunistic infections after initiation of ART and of these, extrapulmonary tuberculosis (29.4%) accounted the largest proportion followed by diarrhea (14.7%) (Table 2).

### Prevalence and trend of treatment failure

Treatment failure was assessed based on the two WHO criteria and from the 269 study participants, 51 (18.96%) failure cases were reported. Majority (12.36%) of the treatment failures were clinical and only 6.6% were immunologic failures. Of total 51 ART failure cases, 12 (23.4%) of them were confirmed by viral load test and switched to second‐line ART regimen. The mean time to develop treatment failure after initiation of first‐line regimen was 41 ± 24.96 months with a range from 16 to 104 months. More than 1 in 10 of the pediatrics developed treatment failure within 24 months. Of those who confirmed to have treatment failure, most (75.0%) of them were switched to second‐line ART in <30 days. After detection of the treatment failure, on average, it took 33.41 ± 11.93 days to initiate the second‐line ARVs regimen (Table 2).

**Figure 1:**
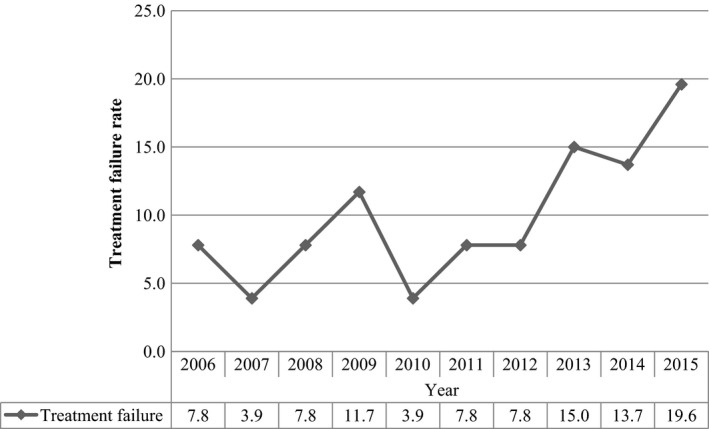
Trend of pediatrics' ART failure in Fiche and Kuyu hospitals followed from 2006 to 2015, North Shewa zone, Oromia region, Ethiopia (*N* = 269).

Trend of pediatrics ART failure was analyzed between January 2006 and December 2015. The result of this study showed that the lowest ART failure rate was reported in 2007 and 2010, while the highest treatment failure was observed in 2015 (Fig. 1).

### Predictors of treatment failure

A logistic regression analysis was conducted to explore the impact of selected sociodemographic variables on pediatric ART failure. Of the nine variables, only age group of 6–9 years and patient status at last visit were associated with treatment failure.

The study found that being in the age of 6–9 years was protective from developing treatment failure (AOR = 0.26, 95% CI: 0.09, 0.72) as compared to 10–15 years old children. In other words, pediatrics in the age group of 6–9 years had a 74% reduction in risk of developing treatment failure than their counterparts. Pediatrics' outcome at last visit was positively associated with treatment failure in that those who lost to follow‐up (AOR = 7.54, 95% CI: 2.35, 24.16), died (AOR = 22.22, 95% CI: 3.75, 131.54), and transferred out (AOR = 3.34, 95% CI: 1.41, 7.87) had more risk of acquiring treatment failure compared to those pediatrics who were still on ART follow‐up (Table 3).

**Table 3:** Sociodemographic variables as a predictor of first‐line ART failure in pediatrics on ART in Fiche and Kuyu hospitals followed from 2006 to 2015, North Shewa zone, Oromia region, Ethiopia (*N* = 269)

Covariates	Treatment success, *N* (%)	Treatment failure, *N* (%)	COR (95% CI)	AOR (95% CI)
Gender				
Female	98 (79.7)	25 (20.3)	1.27 (0.96–2.34)	1.32 (0.64–2.73)
Male	120 (82.2)	26 (17.8)	1.00	1.00
Age (in years)				
<5	30 (88.2)	4 (11.8)	0.37 (0.12–1.13)	0.20 (0.41–1.04)
6–9	75 (91.5)	7 (8.5)	0.26 (0.11–0.62)*	0.26 (0.09–0.72)^a^
10–15	113 (73.9)	40 (26.1)	1.00	1.00
Primary caretaker				
Mother	148 (80.0)	37 (20.0)	1.00	1.00
Father	29 (87.9)	4 (12.1)	0.55 (0.18–1.66)	0.32 (0.84–1.26)
Guardian/relative	41 (80.4)	10 (19.6)	0.97 (0.44–2.12)	0.42 (0.10–1.63)
Employment status of primary caretaker				
Employed	81 (75.7)	26 (24.3)	1.00	1.00
Not employed	137 (84.6)	25 (15.4)	0.56 (0.30–1.05)	0.67 (0.32–1.43)
Mother's HIV status				
Positive	197 (80.7)	47 (19.3)	1.00	1.00
Unknown	21 (84.0)	4 (16.0)	0.79 (0.26–2.43)	0.69 (0.17–2.72)
School enrollment				
Enrolled	162 (79.0)	43 (21.0)	1.00	1.00
Not enrolled	56 (87.5)	8 (12.5)	1.85 (0.82–4.19)	0.87 (0.27–2.86)
Parent status				
Both parent alive	128 (86.5)	20 (13.5)	1.00	1.00
One parent alive	52 (73.2)	19 (26.8)	2.3 (1.15–4.73)	1.82 (0.78–4.21)
Both parents died	38 (76.0)	12 (24.0)	2.0 (0.9–4.5)	2.78 (0.70–11.00)
Status at last visit				
Lost	10 (52.6)	9 (47.4)	8.0 (2.9–22.0)^a^	7.54 (2.35–24.16)^a^
Dead	3 (33.3)	6 (66.7)	17.8 (4.1–76.7)^a^	22.22 (3.75–131.54)^a^
Transferred out	27 (64.3)	15 (35.7)	5.2 (2.4–11.4)^a^	3.34 (1.41–7.87)^a^
On follow‐up	178 (89.4)	21 (10.6)	1.00	1.00
Exposure to NVP				
Yes	204 (80.6)	49 (19.4)	0.95 (0.13–2.70)	1.55 (0.26–9.12)
No	14 (87.5)	2 (12.5)	1.00	1.00
Disclosure status				
Yes	41 (89.1)	5 (10.9)	1.00	1.00
No	177 (79.4)	46 (20.6)	2.13 (0.79–5.69)	2.4 (0.811–7.44)

a Significant at *P* < 0.05.

Clinical and medical variables were also examined for possible association with ART failure and the result showed that study participants who had suboptimal adherence to ART were 4.85 times more likely to develop treatment failure when compared to patient who had optimal adherence (AOR = 4.85, 95% CI: 1.82, 12.93). Children who had baseline CD4 counts <50 cells/mm^3^ were 4.28 times more likely to develop treatment failure when compared to those who had initial CD4 count ≥50 cells/mm^3^ (AOR = 34.2, 95% CI: 3.49, 335.9). Children who were in advanced WHO clinical stage 3 or 4 had 2.56 times more risk of developing treatment failure when compared to those asymptomatic and WHO stage 2 patients (AOR = 2.56, 95% CI: 1.02, 6.45) (Table 4).

**Table 4:** Clinical and treatment data of children's with HIV/AIDS treated with ART from 2006 to 2015 as predictor of ART failure in Fiche and Kuyu hospitals, North Shewa, Oromia, Ethiopia**.**

Covariate	Treatment success, *N* (%)	Treatment failure, *N* (%)	COR (95% CI)	AOR (95% CI)
OI after 6 months ART initiation				
Present	187 (81.3)	43 (18.7)	1.12 (0.48–2.61)	0.97 (0.38–2.46)
Absent	31 (79.5)	8 (20.5)	1.00	1.00
Adherence to ART				
Suboptimal	194 (85.5)	33 (14.5)	4.40 (2.15–9.00)^a^	4.85 (1.82–12.93)^a^
Optimal	24 (57.1)	18 (42.9)	1.00	1.00
Initial WHO stage				
Stage 1 or 2	100 (89.3)	12 (10.7)	1.00	1.00
Stage 3 or 4	118 (75.2)	39 (24.8)	2.75 (1.36–5.54)^a^	2.56 (1.02–6.45)^a^
Initial ARV substitution				
No	115 (83.3)	23 (16.7)	1.00	1.00
Yes	103 (78.6)	28 (21.4)	1.35 (0.73–2.5)	0.87 (0.38–1.99)
Baseline CD4 (*n* = 246)				
<50 cells/mm^3^	2 (25.0)	6 (75.0)	4.60 (2.65–6.71)^a^	4.28 (3.49–5.9)^a^
≥50 cells/mm^3^	195 (81.9)	43 (18.1)	1.00	1.00
Severe malnutrition (wt/age) (*n* = 208)				
Present	77 (82.8)	16 (17.2)	0.93 (0.45–1.90)	0.99 (0.43–2.27)
Absent	94 (81.7)	21 (18.3)	1.00	1.00
Child take IPT				
Yes	139 (88.0)	19 (12.0)	1.00	1.00
No	79 (71.2)	32 (28.8)	2.96 (1.57–5.57)^a^	2.09 (0.88–4.97)

a Significant at *P* < 0.05.

## Discussion

This retrospective cohort study was designed to determine the magnitude and predictors of first‐line ART failure among pediatric patients in Fiche and Kuyu hospitals in Oromia region, Ethiopia. The study showed that clinical failure was commonly encountered than immunologic failure with a total ART failure rate of 18.9%. The proportion of patients that failed treatment in the current study is higher than the retrospective cohort study done in Jimma University hospital and four referral hospitals in Addis Ababa (Workneh et al. 2009; Bacha et al. 2012). The study does not include virologic failures as viral load testing is not routinely available in practice; hence, this figure could underestimate a greater impact of virological failure. This treatment failure magnitude which considers the two WHO criteria, however, is even high and alerts that there should be cautious and frequent monitoring and evaluation of the ART outcomes by health care providers.

On the contrary, the proportion of patients that failed treatment in this study is lower compared to a study conducted in Uganda and Mozambique in 2014, which found that 29% of the patients experienced treatment failure (Costenaro et al. 2015). This could be because in the present study, the system might be failed to timely identify and detect ART failure cases unlike the two different African countries. This also seems to be evident that the mean time to occurrence of treatment failure after initiation of first‐line regimen is 41 ± 24.96 months, which is more than the study done in Malawi (Buck et al. 2010) and South Africa (Davis et al. 2011). Health care providers' lack of knowledge and skill to identify early evidence of treatment failure, frequent machine failure to do CD4, and viral load testing service to support diagnosis could be factors contributing for the delay. Failure of the health care system to detect and switch treatment to second‐line regimen for such patients will worsen the final outcome of HIV patients.

Trend of treatment failure appears to be like a wave with variable amplitude. However, the trend in general showed an increasing rate in treatment failure. Although it needs further investigation, we suspect that either the frequent mentorships and trainings given to ART providers might improve detection of cases or problems like poor adherence increase failure cases.

The current study was in concordance with previous study done in four referral hospitals in Addis Ababa and India (Prabhakar et al. 2011; Bacha et al. 2012) that initiation of ART at advanced immunologic stage CD4 count <50 cell/mm^3^ and advanced WHO clinical stages 3 and 4 are identified as predictors of treatment failure (Workneh et al. 2009). Another study conducted in Mozambique showed that starting ART at later stages of WHO (stage 3 or 4) had a risk of poor outcomes and as a result it recommended initiation of ART at early stages (Andrew et al. 2011).

With regard to age, a study conducted by Bacha et al. (2012) reported that children who were <3 years old were found to be at higher risk of treatment failure. However, this study found that only age between 6 and 9 years was associated with treatment failure and being in this age group was found to have a protective effect. This difference might be seen as pediatrics within 6–9 years age group are assumed to be relatively matured than the <3 years old children, and thus they might take their treatment without or with little support from their parents/caregivers. Thus, those relatively younger children might have lower adherence rate which might expose them to developing ART failure (Bacha et al. 2012).

The success of ART hinges on patient's adherence to their treatment and the present finding revealed that adherence is significantly predictive of ART failure. In this study, patient who had suboptimal adherence to their treatment had 4.85 times more risk of developing treatment failure which mirrors with other studies conducted in Nigeria, Tanzania, and Ethiopia (Ramadhani et al. 2007; Ugwu and Eneh 2013; Yimer and Yalew 2015). Another study in the public facility revealed that the risk factors for treatment failure include nonadherence and missed appointments (Gregory et al. 2007). Consistent to this, the current study showed that more than a quarter (23.4%) of the patients missed more than 30 days of appointment. Besides, lost to follow‐up was found to be predictor of treatment failure where those patients with lost to follow‐up status had 3.34 times more risk of developing treatment failure compared to those who were still on ART follow‐up. This all implies tracing of lost patient from ART clinic need to be strengthened using proper appointment calendar utilization, frequent updating of patient contact address to decreasing lost patients.

The study found that only small proportion (5.9%) of the children were disclosed about their serology status, while their mean age lies between 10 and 14 years which is near to adolescent age. On the other hand, disclosure is not significantly associated with treatment failure which is against other findings from Ethiopia and Tanzania (Ramadhani et al. 2007; Yimer and Yalew 2015). A study done by Yimer and Yalew (2015) revealed that disclosure of HIV infection status to the children was a risk factor for treatment failure mainly due to nonadherence as a result of stigma and discrimination after disclosure. On the other hand, a study conducted by Ramadhani et al. (2007) in Tanzania found that disclosure was protective against treatment failure. Hence, further in‐depth study is needed to correctly identify the effect of disclosure on treatment failure.

Only 5.9% of children have documented NVP prophylaxis provision which is higher than a study conducted by Bacha et al. (2012) in Ethiopia which was found to be 2.4%. However, the proportion of children who received prophylaxis is still lower as there has been remarkable progress in rate of institutional delivery where PMTCT services are freely available. Based on the recent national data, health facility delivery in urban areas including the study area was 59% (Federal Democratic Republic of Ethiopia Central Statistical Agency, 2014). Hence, as most of the pediatrics HIV infection can be prevented by providing standard PMTCT service, and attention should be given to strengthen such services.

## Conclusion

Prevalence of ART failure in children with HIV infection was 18.8% and majority (12.2%) of them were clinical failures. The mean time to ART failure was 41 ± 24.96 months and the mean days to initiation of second‐line ARV after detection of first‐line treatment failure was 33.41 ± 11.93 days. Patients' age between 6 and 9 years is protective from treatment failure, while initiation of ART at low CD4 count <50 cells/mm^3^ and advanced WHO clinical stage 3 or 4, suboptimal adherence, last ART follow‐up outcome reported as lost, dead, transferred out are predictors of treatment failure.

## Authors' Contributions

All authors contributed equally in the design, data collection, and write up of this article. All the authors have read and approved the final manuscript.

## Disclosure

We confirm that this work is original and has not been published elsewhere nor is it currently under consideration for publication elsewhere. The authors have no support or funding to report. The authors have declared that no conflicts of interest exist.
